# Currently monitored microplastics pose negligible ecological risk to the global ocean

**DOI:** 10.1038/s41598-020-79304-z

**Published:** 2020-12-17

**Authors:** Ricardo Beiras, Alexandre M. Schönemann

**Affiliations:** 1 Department of Ecology and Animal Biology, Faculty of Marine Sciences, University of Vigo, 6 36310 Vigo, Galicia, Spain; 2 Department of Biochemistry, Genetics and Immunology, Faculty of Biology, University of Vigo, 36310 Vigo, Galicia, Spain; 3 ECIMAT-CIM, University of Vigo, Illa de Toralla, 36331 Vigo, Galicia, Spain

**Keywords:** Environmental impact, Ecology

## Abstract

Given the rise in plastic production, microplastics (MP) dominate marine debris, and their impact on marine ecosystems will likely increase. However a global quantitative assessment of this risk is still lacking. We conducted an ecological risk assessment of MP in the global ocean by comparing the thresholds of biological effects with the probability of exposure to those concentrations, according to plastic density data adjusted to a log-normal distribution. Levels of MP from 100 to 5000 µm span from < 0.0001 to 1.89 mg/L, whereas the most conservative safe concentration is 13.8 mg/L, and probability of exposure is *p* = 0.00004. Therefore large MP pose negligible global risk. However, MP bioavailability, translocation and toxicity increase as size decreases, and particles < 10 µm are not identified by current monitoring methods. Future research should target the lowest size fractions of MP and nanoplastics, and use in toxicity testing environmental plastic particles rather than engineered materials.

## Introduction

Inappropriate disposal of solid waste makes the global ocean a sink of plastics, which under environmental conditions fragment into smaller pieces termed microplastics (MP) when they become < 5 mm, the most concerning form of ocean plastic debris^1,2^. When the issue of MP accumulation in marine compartments emerged in the scientific literature, concern on harmful effects on marine organisms prompted experimental approaches using very high loads of engineered materials such as polystyrene (PS) microspheres, frequently labeled with fluorescent probes^3–6^. These experiments supported remarkable effects, from reduced ingestion and energy budget to reduced growth of the organisms or their progeny. On that basis, and despite the lack of effects observed in similar reports^7–10^, and criticism on experimental design of laboratory toxicity testing^11–13^, MP are repeatedly considered in the scientific literature as a cause of high concern for marine ecosystem health.

In this review we conduct an ecological risk assessment (ERA) of MP in the global ocean according to the standard methodology derived from the National Academy of Sciences paradigm^14,15^ adapted to the marine environment^16^. According to the paradigm, risk was characterized by comparing the predicted exposure, derived from the data on MP density in the sea, with the thresholds of toxicity found in laboratory toxicity tests using marine organisms. Unlike previous attempts to assess risk posed by MP^13,17^, we have considered in our analysis particle size in order to account for the large differences in impact described across the broad range of sizes from 5 mm down to < 1 µm. This allows refinement of ERA and derivation of environmental quality criteria^18,19^ by introducing in these quantitative estimates plastic particle size as an explanatory variable.

## Results

### Plastic density in the sea follows log-normal distributions

Tables 1 and S1 compile the density of plastics in surface waters of the global ocean. Reported mean densities of plastics in surface waters ranged from 0.12 to 7250 × 10^−3^ Pieces/m^3^, and from 0.01 to 34.09 × 10^−3^ mg/L. Similar amounts, in mass units, have been found in subsurface waters (Table 2). Either recorded in numbers or mass units, plastic density frequently shows a log-normal distribution (see Table 1). The smallest dataset^20^ is normally distributed, and 13 of the remaining 16 datasets fit to log-normal distributions. The exceptions are the three largest datasets^1,21,22^. In the two large datasets by Law et al.^1,21^, repetition of the same values in trawls with low abundance of plastics causes non-normality (see Fig. S1a,b). The dataset reported by Goldstein et al.^22^ has been gathered from different oceanographic cruises, including areas of MP accumulation such as the North Pacific Subtropical Gyre, and areas with lower MP density such as the Eastern Tropical Pacific or Alaska, and the distribution was bimodal, with a Bimodality Coefficient, BC = 0.621 (Fig. S2, in mass units). The variance in plastic density is large and similar in all datasets, with an average coefficient of variation of 201%.

**Table 1:** Plastic density in surface waters; studies for which data from individual trawls were available.

Study	Location	n	10^–3^ Particles/m3				µg/L			
			Mean	Std/CV	Min–max	Log-normal	Mean	Std/CV	Min–max	Log-normal
^23^	N Atlantic	11	9.006	9.224/1.02	0.120–30.761	Yes	0.730	1.293/1.77	0.0015–4.509	Yes
^21^	N Atlantic	3803*	42.266	114.801/2.72	0.676–2308.856	No (*p* < 0.001)	0.575	1.561/2.72	0.009–31.400	No (*p* < 0.001)
^24^	Arctic/N Pacific	157*	94.185	266.034/2.83	2.2–3140.9	Yes	0.131	0.337/2.77	0. 0001–2.5191	Yes
^22^	Pacific	196	2612.694	4855.95/1.86	1–32,765	No (bimodal)	4.620	8.317/1.80	0.001–52.969	No (bimodal)
^25^	Mediterranean	36*	1296.67	1983.49/1.53	100–8920	Yes	2.258	3.454/1.53	0.17–22.8	Yes
^26^	Australia	136*	31.481	46.47/1.48	2.627–287.621	Yes	1.939	2.863/1.48	0.16–17.72	Yes
^27^	N Pacific	1058*	217.590	1654.47/7.60	1.028–49267.784	No (*p* < 0.001)	2.959	22.501/7.60	0.014–670.041	No (*p* < 0.001)
^28^	Mediterranean	28*	421.732	848.43/2.01	12.5–3437.5	Yes	6.981	14.046/2.01	0.21–56.91	Yes
^20^	Mediterranean	10	168.00	103.58/0.62	10–350	Normal	1.374	0.848/0.62	0.08–2.86	Normal
^29^	Mediterranean	39	1219.27	N.m	N.m	–	2.610	3.786/1.45	0.0025–18.11	Yes
^30^	Mediterranean	22*	208.182	347.15/1.67	40–1690	Yes	1.704	2.841/1.67	0.33–13.83	Yes
^31^	Mediterranean	33	1305.869	1312.77/1.01	215.98–5783.77	Yes	5.071	5.097/1.01	0.84–22.46	Yes
^32^	Mediterranean	74/71*	1002.568	1842.62/1.84	20–11300	Yes	1.751	3.926/2.24	0.011–26.081	Yes
^33^	Southern Ocean	5	31	41.9/1.35	99	Yes	N.m	N.m	N.m	–
^34^	Arctic	21*	534.414	706.945/1.32	5.907–2458.07	Yes	0.652	0.975/1.50	0.004–3.524	Yes
^35^	Australia	22	195	135.25/0.70	40–470	Yes	0.814	0.569/0.70	0.2–2.0	Yes
^36^	Arctic	13	128	107/0.84	18–310	Yes	N.m	N.m	N.m	–
^37^	N Atlantic	28	180	205/1.14	35–870	Yes	0.669	0.556/0.83	0.084/2.525	Yes

When data are reported per sea surface area only sampled volume was calculated from the area of the sampling device trawled inside the water. n, sample size; Std/CV, standard deviation/Coefficient of variation; and n.m.,not measured or unavailable data. *Zero values excluded.

**Table 2:** Plastic density in sub-surface waters.

Ocean	*Cruise/region*	Depth (m)	min (µm)	n	Particles/m^3^			µg/L	
					Mean	*Std*	Max	Mean	Max
^38^Atlantic	*Equatorial 2003–04*	0.6	300	88	**0.01**	n. m	n. m	n. m	n. m
^39^N Pacific	*NE Pacific 2012*	4.5	62.5	34	**2080**	***2190***	**9180**	64.9	286
^40^N Atlantic	*IR & UK 2013*	3	250	470	**2.46**^**†**^	***2.43***	**22.5**	0.71	6.53
^41^Arctic	*Barents Sea 2014*	6	250	75	**2.68**^**†**^	***2.95***	**11.5**	0.51	2.17
^42^N Atlantic	*2014*	3	10	23	**148**	***131***	**501**	0.066	0.223
^43^Arctic	*2005 & 2014*	0–50	500	38	**1.72**^**†**^	***0.93***	**4.52**	N. m	N. m
^44^N Pacific	*Off California 2017*	5–1000	100	17	**4.6**	***3.1***	**11**	N. m	N. m
^45^Atlantic	AMT26 JR16001 2016	10–240	25	36	**2782**	***3393***	***11,693***	0.997	2.895
^46^Arctic	*West Greenland 2019*	5	10	6	**142**	***85.83***	278	0.029	0.051

When the studies report data for particles < 5 mm (microplastics) separately these values are shown here. Bold indicates measured values. Mass estimates were obtained from particle size data assuming density = 1, cylindrical shape for fibers and spherical shape otherwise. n.m. not measured or unavailable data. ^†^> 95% fibers.

**Figure 1:**
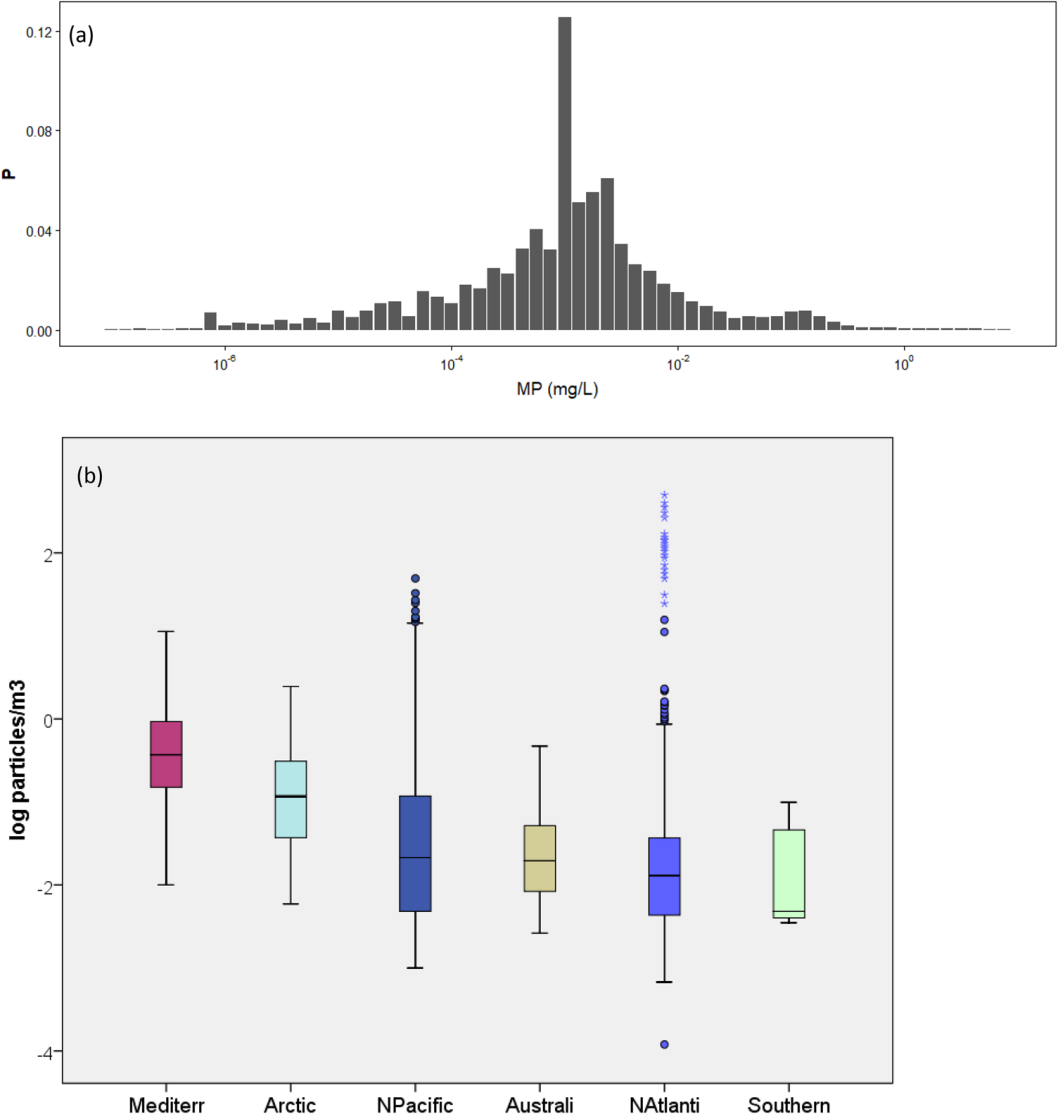
(**a**) Probability (P) distribution of microplastic density (MP, mg/L in log scale) in seawater from all data reviewed. The 0 mg/L values were excluded. (**b**) MP density (log particles/m^3^) in the different oceans sampled ordered from left to right by decreasing median. Horizontal line: median; box boundaries: 25th and 75th percentiles; bars: range of observed values; dots and asterisks: potential outliers.

When all surface plastic density data here reviewed are combined a bell shaped distribution is obtained (Fig. 1a).

### Plastics accumulate in the convergence zones of the subtropical gyres

Median values for plastic debris in surface water found in the present review are similar for all oceans (Fig. 1b, in numbers); in mass units 0.16 µg/L in N Pacific, 0.17 µg/L in the Arctic, and 0.18 µg/L in the North Atlantic. Values are higher in the Australian and Mediterranean data-sets, more influenced by costal sampling sites: 0.92 µg/L for Australia (including both the Indic and Pacific coasts), and 1.02 µg/L for the Mediterranean. On the basis of both original and previously published data gathered from cruises across the global ocean, Cózar et al.^47^ identified zones of accumulation of floating plastic debris in the convergence zones of the five subtropical gyres (N and S Atlantic, N and S Pacific, and Indian oceans), with similar densities ranging from 200 to 500 g/km^2^ across each of the five zones, equivalent to 0.8–2 µg/L. In contrast, mean plastic densities in nonaccumulation zones never exceeded 50 g/km^2^, equivalent to 0.2 µg/L. This means that plastic pollution is a global issue, and position within a particular ocean rather than the ocean itself is more useful to predict plastic density. For example, Law et al.^21^ found a dramatic increase in plastic density in the N Atlantic within a strip at latitudes between 27 and 36°N compared to sites further North or South.

In coastal regions, plastic densities show very high variability, with largely above average at certain specific areas influenced by riverine discharges such as the Yngtze estuary^48^, or surface currents such as Kuroshio^49^.

Concerning variability within sites, rough wind conditions are reported to decrease floating plastic density^25,26,32^, as expected in vertically mixed compared to stratified water columns^50^.

### Toxicity of plastic particles tends to increase as particle size decreases

**Figure 2:**
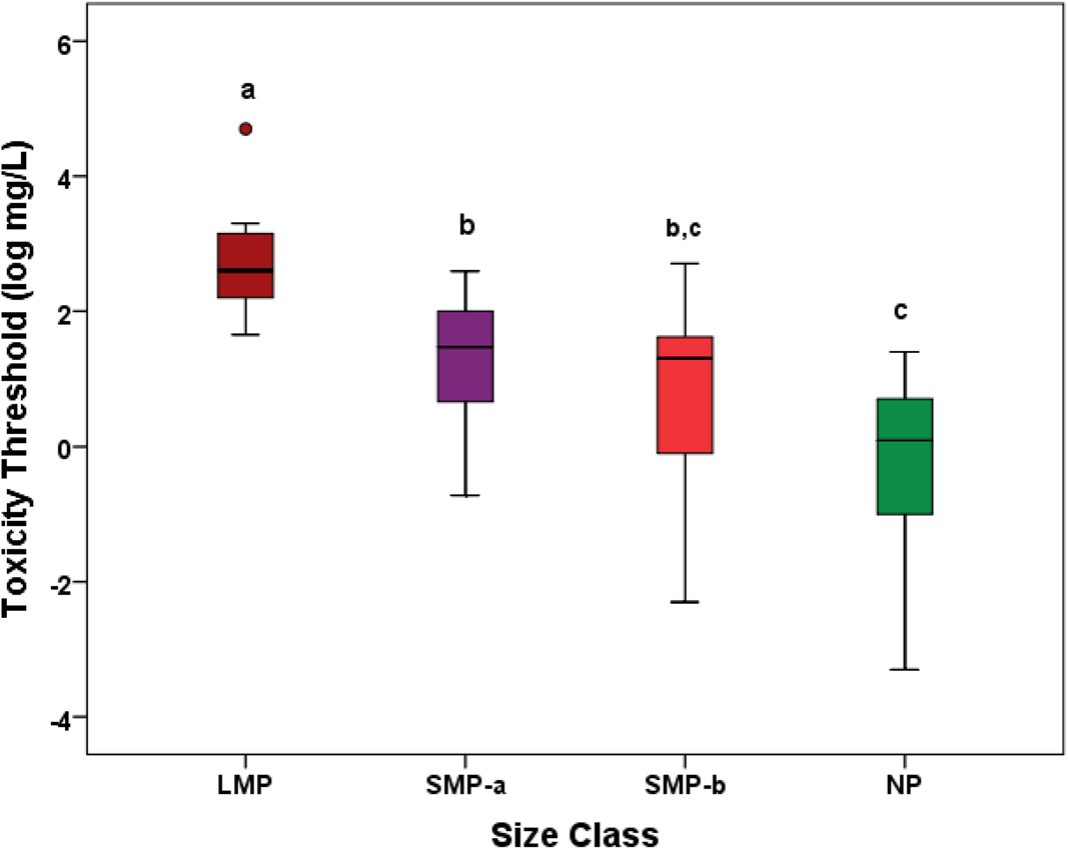
Toxicity thresholds of plastic particles on aquatic organisms. Note the reduction in toxicity threshold, i.e. the increase in toxicity, as particle size decreases. Size classes were defined as: large microplastics (LMP, > 100 µm), small microplastics between > 10 and 100 µm (SMP-a), small microplastics between 1 and 10 µm (SMP-b), and nanoplastics (NP, < 1 µm). Horizontal line: median; box boundaries: 25th and 75th percentiles; bars: range of observed values. LMP shows a potential outlier. Different letters indicate significantly different groups (Tukey, *p* < 0.05).

The effect of particle size on plastic toxicity was investigated in the dataset obtained from the selected studies compiled in Table S1. Toxicity thresholds (TT, mg/L) increased as particle size (S, µm) increased according to a double-logarithmic regression model (*p* < 0.001, n = 68):

$$\log \;{\text{TT}} = 0.297 + 0.739 \cdot \log \;{\text{S}}$$

where the slope is significantly > 0 (95% CI: 0.447–1.031). However, the heterogeneity of the biological models and plastic materials used in laboratory toxicity testing were responsible for a low value of r^2^ = 0.28. When the different plastic particles used in all studies here selected are classified into four size classes, large microplastics (LMP, > 100 µm), small microplastics between > 10 and 100 µm (SMP-a), small microplastics between 1 and 10 µm (SMP-b), and nanoplastics (NP, < 1 µm), then a statistically significant (*p* < 0.0001) trend towards increasing toxicity (lower toxicity thresholds) with decreasing particle size can be observed (Fig. 2). Mean TT values are: 7684, 74.1, 58.9, and 4.9 mg/L respectively.

**Figure 3:**
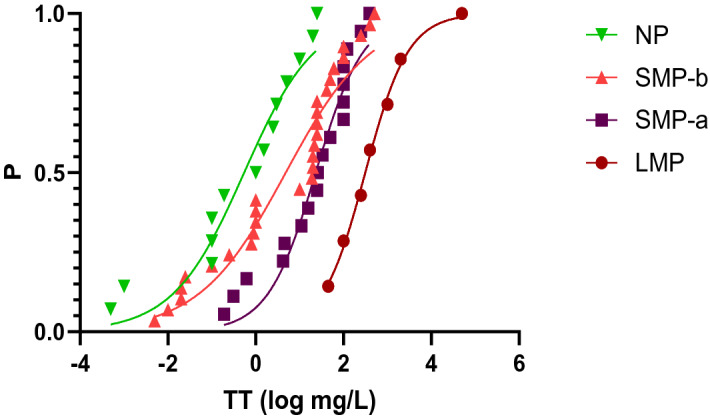
Species sensitivity distribution (SSD) curves fit to the four plastic-size classes, defined as in Fig. 2. Notice lower toxicity thresholds (TT), i.e. higher toxicity, as particle size decreases.

When TT within each size class are fit to species sensitivity distribution (SSD) curves, data fit well to log-logistic functions (Fig. 3, Table 3), allowing calculation of the 5th percentile (HC_5_) for the four size classes. HC_5_ values are all significantly different among them and, as expected, toxicity increases as particle size decreases. The HC_5_ values are similar in magnitude to the critical values (Table 3), i.e. the lowest concentration for each size class ever found to cause a significant biological effect according to the literature.

**Table 3:** Fitting parameters of SSD curves using log-logistic functions (see Material and methods) for diferent plastic particle size classes: large microplastics (LMP, > 100 μm), small microplastics between 10 and 100 μm (SMP-a), small microplastics between 1 and 10 μm (SMP-b), and nanoplastics (NP, < 1 μm).

Size class	a (mg/L)	b	n	AIC	HC_5_ (mg/L)	Critical value (mg/L)	Critical value endpoint
LMP	468 (388–548)	1.13 (0.94–1.33)	7	− 34.95	16.68 (13.82–19.54)	44.7	*Gammarus fossarum* growth
SMP-a	28.01 (23.22–32.80)	1.22 (1.03–1.41)	18	− 95.69	0.76 (0.63–0.89)	0.19	*Sebastes schlegelii* growth
SMP-b	6.00 (4.23–7.78)	2.23 (1.92–2.55)	29	− 145.72	0.008 (0.006–0.01)	0.01	*Crassostrea gigas* larval growth of progeny
NP	0.77 (0.54–1.00)	1.84 (1.52–2.16)	14	− 72.15	0.003 (0.002–0.004)	0.0005	*C. gigas* larval ingestion rate

### Large-size MP currently pose a negligible ecological risk across the global ocean

Risk posed by MP within the range of sizes normally monitored in oceanographic cruises (LMP) can be characterized by obtaining from the SSD curve the maximum levels considered safe in environmental management. This may be estimated from the HC_5_, which would protect 95% of the species in the ecosystem, or more conservatively by the lower end of its 95% CI, which would ensure that protection with a certainty > 95%. The corresponding values are 16.7 and 13.8 mg/L respectively (see Table 3), one order of magnitude above the single highest MP density concentration ever reported in the scientific literature (1.89 mg/L)^51^, and ca. 2 orders of magnitude above the highest mean value of all datasets (0.32 mg/L^52^, for S Portuguese coast).

In addition, risk can be more formally characterized by assessing the probability of exposure of marine organisms to MP concentrations above the safe value of 13.8 mg/L. According to the global MP distribution in marine waters (see Fig. 1) this probability is 0.00004. Therefore, on the basis of the current knowledge on environmental levels, and their toxicity quantified in laboratory experiments, the risk posed by LMP is negligible across the whole global ocean, including central oceanic gyres and coastal hot spots.

### Risk assessment for small-size MP

Very limited information is available in the scientific literature on the plastic density for particles within the 10–100 µm range SMP-a, and none for smaller particles. The only study found entirely covering this range of particles gathers data from 23 samples taken by the vessel’s underway intake and filtered by 10 µm mesh^42^. The abundance of plastic particles within this range of SMP found in that study fits well with the comprehensive distribution of abundance vs particle size reported by Isobe et al.^51^ (See Fig. S3), and the size distribution is consistent with that reported using a slightly higher threshold of minimum particle size^45^. When data from those studies are pooled together in a single distribution of abundances of particles ranging from 10 to 5000 µm and abundances are expressed in mass units, then the proportion represented by size class SMP-a is just 0.023%. Pabortsava and Lampitt^45^ though reported a 15-fold higher mass of MP than Enders et al.^42^, resulting in a conservative estimation of 0.35% of SMP-a size MP, in mass. Assuming the distribution across the global ocean (see Fig. 1) is also representative for SMP-a, the resulting risk coefficient, quantified by the probability of exposure to SMP concentrations above the safe value obtained for SMP-a, is even lower than that for LMP (*p* = 2.28 × 10^−7^).

## Discussion

Currently MP risk assessment is hampered by the mismatch between the size range of MP recorded in the marine environment (frequently > 0.3 mm) and those reported as posing risk to marine organisms in laboratory toxicity tests (frequently < 20 µm). With these limitations in mind, and on the basis of the current knowledge of MP abundance and effects, the probability for a marine organism to be exposed to concentrations above those considered safe is *p* = 0.00004. Therefore, the risk posed to marine ecosystems by plastic particles within the size range currently monitored by standard methods can be classified as negligible. This is in line with previous assessments^12,53^.

However, increasing scientific evidence supports that, disregarding biological model, plastic particle size affects their biological effects. The present study found by regression analysis that the logarithm of the toxicity threshold increases with a positive slope (b = 0.784; 95% CI: 0.475–1.093) as the logarithm of particle size increases (*p* < 0.001, n = 62). Moreover, when plastic particles used in toxicity testing were classified into 4 size classes, the effect of particle size on toxicity was highly significant (ANOVA, *p* < 0.0001), and the dependence of safe concentration (HC_5_) on particle size was even more remarkable, with a slope b = 1.21 according to the equation:

$$\log \;{\text{HC}}_{5} = 1.21\;\log \;{\text{S}}{-}2.10$$

where S is the mean particle size (µm) for each size class (r^2^ = 0.92, n = 4).

Therefore, the plastic particles with the highest ecological impact are expected to be below the threshold of sizes captured by current standard methodologies of oceanographic sampling. To the best of our knowledge, only four studies attempted sampling of plastic particles below 100 µm size (see Table 2), and only three of them confirmed the plastic nature of the particles found (by using Raman micro-spectrometry^42^ and linear-array FTIR imaging System). These data allow estimates of SMP-a loads in the water between 0.029 and 2.9 µg/L. These figures are again well below the safe concentration for 10–100 µm MP (0.35 mg/L), and even below that for the 1–10 µm size class (0.005 mg/L) (see Table S2). In conclusion, the scarce data available point also at low ecological risk associated to those small-size MP.

Another remarkable limitation of the ecotoxicological dataset is that only 2 from the 68 TT values here compiled correspond to MP from environmental sources. The other studies work with engineered particles or industrial products that may not be fully representative of secondary MP weathered in the environment. Frequently the tested materials are made of PS, which is not among the three most abundant polymers in the sea^54^, and carry fluorescent chemicals or other dyes intended to facilitate studies of particle ingestion and distribution throughout the organism. Dispersants and preservatives are also common in commercial PS beads. The six most toxic particles, showing TT < 0.1 mg/L in ecotoxicological studies, all corresponded with particles labelled with fluorescent dyes. However, mean TT of dyed versus virgin particles were similar, and no significant differences in toxicity were found between unlabelled and dye-labelled MP (*p* = 0.15; two-way ANOVA with size class and dye as factors). Future research should focus on the toxicity of MP from environmental sources, since their shape and composition is very different from engineered particles, and thus provide estimates of toxicity more environmentally relevant than artificial materials. In fact, plastic fragments may have a more abrasive surface geometry and cause more physical damage than microspheres. Despite previous identification of the use of PS and other engineered microbeads as a limitation for the environmental relevance of laboratory tests with MP^11,13^, this microbeads continuous to be used and published.

Various mechanisms contribute to explain the higher impact of smaller particles. Most zooplankton and benthic filter feeders preferably ingest particles between 1 and 20 µm. Jeong et al.^55^ proved that 0.1 µm and 2 µm PS particles were both readily ingested by a marine copepod, but 2 µm particles were more easily egested than 0.1 µm particles, which remained—although at low amounts- in the organism even in the presence of food. Fernández and Albentosa^56^ using marine mussels showed that uptake and residence time in the organism increased as particle size decreased. However, this trend may not hold for smaller size ranges, since Al-Sid-Cheikh et al.^57^ modeling uptake and depuration of 24 nm versus 150 nm particles in scallops, reached the opposite conclusion. This is consistent with a lower threshold for efficient particle capture by filter feeders. There is an upper limit in size for translocation of plastic particles from the digestive system to other tissues, although the value for this limit is an object of debate^58^. Finally, environmental fragmentation of both polyoleofins and biopolymers into plastic particles < 1 µm that can diffuse across biological membranes have been reported to account for enhanced deleterious effects on aquatic organisms^59,60^.

Future field surveys should address plastics < 10 µm, the most toxic and bioavailable fraction for which environmental levels are unknown. Standard methods for monitoring the abundance of this low size ranges are urgently needed in order to conduct a more accurate assessment of the risk posed by plastic pollution in the global ocean.

## Methods

### Data acquisition

A comprehensive review was conducted on the available scientific data on, first, plastic particle density in surface and subsurface waters of the global ocean, and, secondly, toxicity of plastic particles to aquatic organisms. Concerning surface plastic densities, Song et al.^61^ reported that different sampling devices produce differences up to 4 orders of magnitude in floating microplastic abundance. Therefore, in order to allow data intercomparability, surface sampling techniques here reviewed were limited to manta trawl or neuston nets, corresponding to the most common sampling procedures. In those devices mesh size used ranged from 200 to 505 µm, with few exceptions^62,63^ that used nylon mesh of ca. 900 µm. Goldstein et al.^22^ reported that 98.5% of the particles caught in a 333 μm mesh would also have been caught in a 505 μm mesh, and there were no significant differences in particle size spectra using one size or another (*p* = 0.22; Kolmogorov Smirnof test).

Common units chosen for this review were pieces per cubic meter for numerical density, and mg/L for mass density. When weight was not recorded but detailed measurements of particle size were available, plastic mass was estimated from particle size assuming 1 g/cm^3^ density, cylindrical shape for fibers and spherical shape otherwise. A number of studies report that most microplastics found in marine waters are fibers^41,64,65^. Due to the lack of data in mass units and difficulties to estimate fiber mass from fiber length data, these studies could not be included in the present risk assessment unless detailed information on particle size distribution in more than one dimension were provided. Unlike fragments, pellets or films, airborne microfibers, for example from laboratory coats, can easily contaminate environmental samples during their analysis, and special precautions advised include working in clean airflow cabinets, normally not available on board of oceanographic vessels, and running filter controls^42^. In fact some studies simply exclude fibers from the records due to the risk of sample contamination^34,37^.

Marine plastic density data in mass (mg/L) units were compiled in a single database to calculate a probabilistic distribution as described in Adam et al.^17^. Briefly, four types of data were found: (I) Studies reporting individual values for every trawl. These data were directly input into the database. (II) Studies reporting mean and standard deviation (s.d.) only. In these cases a log-normal distribution (see below) was modeled using the reported mean and sd as parameters. (III) Studies reporting mean and no s.d. In these cases dispersion was modeled using the mean and the average coefficient of variation of all datasets (201%, see Table 1). The probability distributions described above were pooled in order to generate a single distribution of microplastic abundance in the global ocean.

Concerning toxicity of plastic particles, an ecotoxicological database was built gathering all peer reviewed reports found on the toxicity of artificial polymer particles from 5 mm down to 0.01 µm. This includes the following size classes: LMP (from 5000 µm down to 100 µm), approximately corresponding to the range of sizes reported in oceanographic cruises using conventional plankton sampling devices, small microplastics, from 100 µm down to 1 µm, divided for this study in subclasses SMP-a (100–10 µm) and SMP-b (10–1 µm), and NP (< 1 µm). From the complete ecotoxicological database (114 papers) a subset reporting endpoints with proved ecological relevance affecting population size, i.e. those related with growth, reproduction and survival, was selected. Effects (both stimulant or inhibitory) on enzymatic activities or gene expression were excluded from this selection. Examples of endpoints not directly connected with ecological fitness are induction of antioxidant enzymes or other exposure biomarkers, histological observations, or minor changes in the biochemical composition of tissues. Examples of endpoints with proved ecological relevance are impairment of embryo-larval development, egg production, growth rate, immobilization or mortality. When the same study reported several ecologically relevant endpoints for the same species, the lowest effective concentration was included in the database. In contrast, when different studies reported the same endpoint in the same organism all values were considered and no attempt was made to judge the more representative testing material, even when values were broadly dissimilar (e.g.^66,67^ for nanoplastics and microalgae, or^9,68^ for small microplastics and bivalves). No attempt was made to judge quality of published data, but studies reporting non-monotonic dose:response curves (where effects do not increase with dose) or showing no effects at the highest concentration tested when that concentration is comparatively low were discarded.

### Statistical methods

The TT reported in the selected studies are compiled in Table S2. Following standard procedures, the cumulative distributions (P) of the TT values, expressed in 260 mg/L, were fit to log-logistic functions according to the equation^69^:

$$P = \frac{1}{{1 + 10^{((\log a - \log TT))/b} }}$$

where *a* corresponds to the median of the TT distribution, and 1/*b* is the slope of the curve. From these distributions, termed in the ecotoxicological literature SSD curves, the HC_5_ and the 95% confidence intervals were calculated. These parameters are commonly used for derivation of water quality criteria according to standard methods^18,19,70,71^. Statistical analyses were conducted using IBM SPSS (v. 23) and R (v 3.5.0) statistical software. Effects of size and presence of dye in the particles on log-transformed TT were analyzed by two-way ANOVA. Normal Distribution and homoscedasticity were tested using the Shapiro–Wilk and Levene tests respectively. Bimodal distribution was tested using the Bimodality Coefficient (BC), according to the expression^72^:

$$BC = \frac{\gamma 2 + 1}{{\kappa + \frac{{3\left( {n - 1} \right)^{2} }}{{\left( {n - 2} \right)\left( {n - 3} \right)}}}}$$

where *γ* is the skewness, *κ* is the excess kurtosis, and *n* is the sample size. BC values above 0.555 correspond to bimodality.

Log-logistic models were fit using *nls.multstart* package^73^ and log-normal models using *fitdistrplus* package^68,74^, both from R^75^.

## Supplementary information

Supplementary Information

## References

[1] Law K, Thompson RC (2014). Microplastics in the seas—concern is rising about widespread contamination of the marine environment by microplastics. Science (80-.).

[2] Amaral-Zettler LA (2015). The biogeography of the plastisphere: implications for policy. Front. Ecol. Environ..

[3] Sussarellu R (2016). Oyster reproduction is affected by exposure to polystyrene microplastics. Proc. Natl. Acad. Sci. U. S. A..

[4] Della Torre C (2014). Accumulation and embryotoxicity of polystyrene nanoparticles at early stage of development of sea urchin embryos Paracentrotus lividus. Environ. Sci. Technol..

[5] Cole M, Galloway TS (2015). Ingestion of nanoplastics and microplastics by pacific oyster larvae. Environ. Sci. Technol..

[6] Cole M (2013). Microplastic ingestion by zooplankton. Environ. Sci. Technol..

[7] Browne MA, Dissanayake A, Galloway TS, Lowe DM, Thompson RC (2008). Ingested microscopic plastic translocates to the circulatory system of the mussel, Mytilus edulis (L.). Environ. Sci. Technol..

[8] Browne MA, Niven SJ, Galloway TS, Rowland SJ, Thompson RC (2013). Microplastic moves pollutants and additives to worms, reducing functions linked to health and biodiversity. Curr. Biol..

[9] Van Cauwenberghe L, Claessens M, Vandegehuchte MB, Janssen CR (2015). Microplastics are taken up by mussels (Mytilus edulis) and lugworms (Arenicola marina) living in natural habitats. Environ. Pollut..

[10] Green DS (2016). Effects of microplastics on European flat oysters, Ostrea edulis and their associated benthic communities. Environ. Pollut..

[11] Lenz R, Enders K, Nielsen TG (2016). Microplastic exposure studies should be environmentally realistic. Proc. Natl. Acad. Sci. U. S. A..

[12] Connors KA, Dyer SD, Belanger SE (2017). Advancing the quality of environmental microplastic research. Environ. Toxicol. Chem..

[13] Burns EE, Boxall ABA (2018). Microplastics in the aquatic environment: evidence for or against adverse impacts and major knowledge gaps. Environ. Toxicol. Chem..

[14] Council, N. R. *Risk Assessment in the Federal Government: managing the process*. (1983). 25032414

[15] Suter II GW (2007). Ecological Risk Assessment.

[16] Beiras, R. *Marine Pollution. Sources, fate and effects of pollutants in coastal ecosystems*. (Elsevier, 2018).

[17] Adam V, Yang T, Nowack B (2019). Toward an ecotoxicological risk assessment of microplastics: comparison of available hazard and exposure data in freshwaters. Environ. Toxicol. Chem..

[18] US EPA. Guidelines for deriving numerical national water quality criteria for the protection of aquatic organisms and their uses. PB85–227049. *Environ. Prot.* 105 (2010).

[19] European Commission. *Common Implementation Strategy for the WFD (2000/60.EC), Guidance Document No . 27, Technical Guidance For Deriving Environmental Quality Standards*. (2011). 10.2779/43816.

[20] de Lucia GA (2014). Amount and distribution of neustonic micro-plastic off the western Sardinian coast (Central-Western Mediterranean Sea). Mar. Environ. Res..

[21] Law KL (2010). Plastic accumulation in the North Atlantic subtropical gyre. Science (80-.).

[22] Goldstein MC, Rosenberg M, Cheng L (2012). Increased oceanic microplastic debris enhances oviposition in an endemic pelagic insect. Biol. Lett..

[23] Carpenter EJ, Smith KL (1972). Plastics on the Sargasso sea surface. Science (80-.).

[24] Doyle MJ, Watson W, Bowlin NM, Sheavly SB (2011). Plastic particles in coastal pelagic ecosystems of the Northeast Pacific ocean. Mar. Environ. Res..

[25] Collignon A (2012). Neustonic microplastic and zooplankton in the North Western Mediterranean Sea. Mar. Pollut. Bull..

[26] Reisser, J. *et al.* Marine plastic pollution in waters around Australia: Characteristics, concentrations, and pathways. *PLoS ONE* **8** (2013). 10.1371/journal.pone.0080466 PMC3842337 24312224

[27] Law KL (2014). Distribution of surface plastic debris in the eastern Pacific ocean from an 11-year data set. Environ. Sci. Technol..

[28] Collignon A, Hecq JH, Galgani F, Collard F, Goffart A (2014). Annual variation in neustonic micro- and meso-plastic particles and zooplankton in the Bay of Calvi (Mediterranean-Corsica). Mar. Pollut. Bull..

[29] Cózar A (2015). Plastic accumulation in the mediterranean sea. PLoS ONE.

[30] Panti C (2015). Occurrence, relative abundance and spatial distribution of microplastics and zooplankton NW of Sardinia in the Pelagos Sanctuary Protected Area Mediterranean Sea. Environ. Chem..

[31] Pedrotti ML (2016). Changes in the floating plastic pollution of the mediterranean sea in relation to the distance to land. PLoS ONE.

[32] Suaria, G. *et al.* The Mediterranean Plastic Soup: Synthetic polymers in Mediterranean surface waters. *Sci. Rep.* **6** (2016). 10.1038/srep37551 PMC5120331 27876837

[33] Isobe A, Uchiyama-Matsumoto K, Uchida K, Tokai T (2017). Microplastics in the Southern Ocean. Mar. Pollut. Bull..

[34] Cózar A (2017). The Arctic Ocean as a dead end for floating plastics in the North Atlantic branch of the Thermohaline Circulation. Sci. Adv..

[35] Jensen LH, Motti CA, Garm AL, Tonin H, Kroon FJ (2019). Sources, distribution and fate of microfibres on the Great Barrier Reef Australia. Sci. Rep..

[36] Mu J (2019). Microplastics abundance and characteristics in surface waters from the Northwest Pacific, the Bering Sea, and the Chukchi Sea. Mar. Pollut. Bull..

[37] Poulain M (2019). Small microplastics as a main contributor to plastic mass balance in the north atlantic subtropical gyre. Environ. Sci. Technol..

[38] Ivar do Sul JA, Costa MF, Barletta M, Cysneiros FJA (2013). Pelagic microplastics around an archipelago of the Equatorial Atlantic. Mar. Pollut. Bull..

[39] Desforges JPW, Galbraith M, Dangerfield N, Ross PS (2014). Widespread distribution of microplastics in subsurface seawater in the NE Pacific Ocean. Mar. Pollut. Bull..

[40] Lusher AL, Burke A, O’Connor I, Officer R (2014). Microplastic pollution in the Northeast Atlantic Ocean: Validated and opportunistic sampling. Mar. Pollut. Bull..

[41] Lusher AL, Tirelli V, O’Connor I, Officer R (2015). Microplastics in Arctic polar waters: The first reported values of particles in surface and sub-surface samples. Sci. Rep..

[42] Enders K, Lenz R, Stedmon CA, Nielsen TG (2015). Abundance, size and polymer composition of marine microplastics ≥10 μm in the Atlantic Ocean and their modelled vertical distribution. Mar. Pollut. Bull..

[43] Amélineau F (2016). Microplastic pollution in the Greenland Sea: background levels and selective contamination of planktivorous diving seabirds. Environ. Pollut..

[44] Choy CA (2019). The vertical distribution and biological transport of marine microplastics across the epipelagic and mesopelagic water column. Sci. Rep..

[45] Pabortsava K, Lampitt RS (2020). High concentrations of plastic hidden beneath the surface of the Atlantic Ocean. Nat. Commun..

[46] Rist S (2020). Quantification of plankton-sized microplastics in a productive coastal Arctic marine ecosystem. Environ. Pollut..

[47] Cózar A (2014). Plastic debris in the open ocean. Proc. Natl. Acad. Sci. U. S. A..

[48] Zhao S, Zhu L, Wang T, Li D (2014). Suspended microplastics in the surface water of the Yangtze Estuary System, China: first observations on occurrence, distribution. Mar. Pollut. Bull..

[49] Yamashita R, Tanimura A (2007). Floating plastic in the Kuroshio Current area, western North Pacific Ocean. Mar. Pollut. Bull..

[50] Kukulka T, Proskurowski G, Morét-Ferguson S, Meyer DW, Law KL (2012). The effect of wind mixing on the vertical distribution of buoyant plastic debris. Geophys. Res. Lett..

[51] Isobe A, Uchida K, Tokai T, Iwasaki S (2015). East Asian seas: a hot spot of pelagic microplastics. Mar. Pollut. Bull..

[52] Frias JPGL, Otero V, Sobral P (2014). Evidence of microplastics in samples of zooplankton from Portuguese coastal waters. Mar. Environ. Res..

[53] Gouin T (2019). Toward the development and application of an environmental risk assessment framework for microplastic. Environ. Toxicol. Chem..

[54] Erni-Cassola G, Zadjelovic V, Gibson MI, Christie-Oleza JA (2019). Distribution of plastic polymer types in the marine environment A meta-analysis. J. Hazard. Mater..

[55] Jeong CB (2017). Adverse effects of microplastics and oxidative stress-induced MAPK/Nrf2 pathway-mediated defense mechanisms in the marine copepod Paracyclopina nana. Sci. Rep..

[56] Fernández B, Albentosa M (2019). Insights into the uptake, elimination and accumulation of microplastics in mussel. Environ. Pollut..

[57] Al-Sid-Cheikh M (2018). Uptake, whole-body distribution, and depuration of nanoplastics by the scallop pecten maximus at environmentally realistic concentrations. Environ. Sci. Technol..

[58] Baumann L, Schmidt-Posthaus H, Segner H, Wolf JC (2016). Comment on ‘uptake and accumulation of polystyrene microplastics in zebrafish (Danio rerio) and toxic effects in liver’. Environ. Sci. Technol..

[59] González-Pleiter M (2019). Secondary nanoplastics released from a biodegradable microplastic severely impact freshwater environments. Environ. Sci. Nano.

[60] Enfrin M (2020). Release of hazardous nanoplastic contaminants due to microplastics fragmentation under shear stress forces. J. Hazard. Mater..

[61] Song YK (2014). Large accumulation of micro-sized synthetic polymer particles in the sea surface microlayer. Environ. Sci. Technol..

[62] Colton JB, Knapp FD, Burns BR (1974). Plastic particles in surface waters of the Northwestern Atlantic. Science (80-.).

[63] Ryan PG (1988). The characteristics and distribution of plastic particles at the sea-surface off the southwestern Cape Province South Africa. Mar. Environ. Res..

[64] Cole M (2014). Isolation of microplastics in biota-rich seawater samples and marine organisms. Sci. Rep..

[65] Beer S, Garm A, Huwer B, Dierking J, Nielsen TG (2018). No increase in marine microplastic concentration over the last three decades—a case study from the Baltic Sea. Sci. Total Environ..

[66] Sjollema SB, Redondo-Hasselerharm P, Leslie HA, Kraak MHS, Vethaak AD (2016). Do plastic particles affect microalgal photosynthesis and growth?. Aquat. Toxicol..

[67] Gambardella C (2018). Ecotoxicological effects of polystyrene microbeads in a battery of marine organisms belonging to different trophic levels. Mar. Environ. Res..

[68] Gardon T, Reisser C, Soyez C, Quillien V, Le Moullac G (2018). Microplastics affect energy balance and gametogenesis in the pearl oyster pinctada margaritifera. Environ. Sci. Technol..

[69] Durán I, Beiras R (2017). Acute water quality criteria for polycyclic aromatic hydrocarbons, pesticides, plastic additives, and 4-Nonylphenol in seawater. Environ. Pollut..

[70] van Straalen NM, Denneman CAJ (1989). Ecotoxicological evaluation of soil quality criteria. Ecotoxicol. Environ. Saf..

[71] SCHEER. *Scientific advice on Guidance document n°27: Technical guidance for deriving environmental quality standards*. *Scientific Committee on Health, Environmental and Emerging Risks* (2017).

[72] Pfister R, Schwarz KA, Janczyk M, Rick Daleand JBF (2013). Good things peak in pairs: a note on the bimodality coefficient. Front. Psychol..

[73] Padfield, D. & Matheson, G. nls.multstart: Robust Non-Linear Regression using AIC Scores. R package version 1.0.0. (2018).

[74] Delignette-Muller ML, Dutang C (2015). fitdistrplus: an R package for fitting distributions. J. Stat. Softw..

[75] R Core Team. R: A language and environment for statistical computing. *R fundation, Vienna Austria* (2019).

