# Supplementary material for: Currently monitored microplastics pose negligible ecological risk to the global ocean
Source: Sci Rep. 2020 Dec 17;10:22281. doi: 10.1038/s41598-020-79304-z (PMC7746749; doi:10.1038/s41598-020-79304-z)
Supplement: Supplementary file 1 — Supplementary Information [file 41598_2020_79304_MOESM1_ESM.docx]

**Currently monitored microplastics pose negligible ecological risk to the global ocean**

Ricardo Beiras, Alexandre M. Schönemann

Table S1: Plastic density in surface waters; studies reporting mean values only. When the studies report data for particles<5 mm (microplastics) separately these values are shown here. When data are reported per sea surface area only sampled volume was calculated from the area of the sampling device trawled inside the water. * zero values excluded; # total for S Pacific; † 90th percentile; ‡ manta trawl data only.

|  |  |  |  | Particles/km^2^ | | | 10^-3^ Particles/m3 | | | g/km2 | | | µg/L |  |  |
| --- | --- | --- | --- | --- | --- | --- | --- | --- | --- | --- | --- | --- | --- | --- | --- |
| study | Ocean | *Cruise/*  *Region* | n | mean | std | max | mean | std | max | mean | std | max | mean | std | max |
| ^1^ | N Atlantic | *Oregon II* | 64 | 60.6 | - | 1292 | 0.12 | *-* | 2.584 | 10.5 | - | 222.1 | 0.02 | - | 0.44 |
|  |  | *Albatross* | 40 | 148.4 | - | 2707 | 0.3 | *-* | 5.414 | 18.1 | - | 214.9 | 0.04 | - | 0.43 |
|  |  | *Delaware* | 143 | 8317.5 | - | 262276 | 16.64 | *-* | 524.552 | 77.7 | - | 1403.5 | 0.16 | - | 2.81 |
| ^2^ | Arctic/N Pacific | *Bering Sea 74-75* | 20 | 68 | - | - | 0.91 |  | - | - | - | - | - |  | *-* |
|  |  | *Bering Sea 85* | 8 | 80 | 190 | - | 0.19 |  | - | 3.0 | 7.1 | - | 0.01 |  | *-* |
|  |  | *Subarctic 76* | 14 | 0 | - | - | 0 |  | - | 0 | - | - | 0.00 |  | *-* |
|  |  | *Subarctic 85* | 22 | 3370 | 2380 | - | 7.96 |  | - | 45.8 | 54.7 | - | 0.11 |  | *-* |
|  |  | *G. Alaska 74-75* | 51 | 132 | - | - | 1.76 |  | - | - | - | - | - |  | *-* |
|  |  | *Subtr. Pacif. 72* | 33 | - | - | - | - |  | - | 300 | - | - | 4.0 |  | *-* |
|  |  | *Subtr. Pacif. 76* | 14 | - | - | - | - |  | - | 100 | - | - | 1.23 |  | *-* |
|  |  | *Subtr. Pacif. 85* | 2 | 96100 | 0.78x10^6^ | - | 226.92 |  | - | 1210 | 11310 | - | 2.86 |  | *-* |
| ^3^ | Arctic/N Pacific | *Bering Sea* | 66 | 100 | 600 | - | 0.62 |  | - | 1.0 | 4.2 | - | 0.01 |  | *-* |
|  |  | *Subarctic* | 64 | 12800 | 22300 | - | 78.95 |  | - | 61.4 | 225.5 | - | 0.38 |  | *-* |
|  |  | *Transitional* | 60 | 57900 | 72800 | - | 357.14 |  | - | 291.6 | 714.4 | - | 1.80 |  | *-* |
|  |  | *Subtropical* | 2 | 61000 | 74000 | - | 376.26 |  | - | 535.1 | 726.1 | - | 3.30 |  | *-* |
|  |  | *Japan* | 11 | 74700 | 73800 | - | 460.76 |  | - | 128.2 | 172.2 | - | 0.79 |  | *-* |
| ^4^ | *S Atlantic* | *S Africa 77-78* | 374* | 3639 | 14633 | 445860 |  |  |  | 42.4 | 476.8 | 10920 | 0.2 |  | 52.0 |
| ^5^ | *N Pacific* | *Central Gyre 99* | 11 | 331809 | - | 969777 | 2210 |  | 6465 | 5114 | - | 30169 | 34.09 |  | 201.13 |
| ^6^ | *N Pacific* | *Long Beach 2000-01* | 5 | *-* | - | *-* | 7250 |  | 20000 | *-* | - | *-* | 2.0 |  | 9.0 |
| ^7^ | *N Pacific* | Japan 2000-01 | 76 | 174000 | 467000 | 3.52*10^6^ | 696 |  | 14080 | 3600 | 18100 | 153000 | 14.4 |  | 612 |
| ^8^ | *N Pacific* | *China 2013* | 15 | - | - | - | 167 | 138 | 455 | - | - | - | 0.71 |  | 1.93 |
| ^9^‡ | *N Pacific* | *Korea 2012* | 20 | - | - | - | 47000 | 192000 | ca. 0.9x10^6^ | - | - | - | 24.61 |  | ca. 500 |
| ^10^ | *S Pacific* | *outside converg.* | 549^#^ | - | - | - | - | - | - | 3.9 | - | 7.7^†^ | 0.02 |  | 0.03^†^ |
|  |  | *converg. zone* |  | - | - | - | - | - | - | 112.2 | - | 282.3^†^ | 0.45 |  | 1.13^†^ |
| ^11^ | *S Atlantic* | *Tropical 2011-13* | 160 | - | - | - | 30 | - | - | - | - | - | 0.2 |  | - |
| ^12^ | *N Atlantic* | *Aveiro* |  | - | - | - | 2 | 1 | - | - | - | - | 10 | 10 | - |
|  |  | *Lisboa* |  | - | - | - | 33 | 21 | - | - | - | - | 60 | 40 | - |
|  |  | *Costa Vicentina* |  | - | - | - | 36 | 27 | - | - | - | - | 70 | 60 | - |
|  |  | *Algarve* |  | - | - | - | 14 | 12 | - | - | - | - | 320 | 300 | - |
| ^13^ | *N Pacific* | *Japan 2014* | 56 | 1.72x10^6^ | - | - | 3740 | 10400 | 0.49x10^6^ | - | - | - | 28.7 |  | 1886 |
| ^14^ | *Mediterr.* | *2011-12* | 41 | 130000 | 91309 | 420000 | 1300 |  | 4200 | 57 | 40.7 | 216 | 0.57 |  | 2.16 |
| ^15^ | *N Atlantic* | *Galicia 2014* | 9 | 285000 | 359000 | - | 2850 | 3590 | - | 33.3 | 37.5 | - | 0.33 |  | - |
|  |  | *Cantabrian 13-14* | 26 | 60500 | 92500 | - | 605 | 925 | - | 39.9 | 63.2 | - | 0.40 |  | - |

Tab. S2. Toxicity thresholds (TT, mg/L) of plastic particles arranged by decreasing size classes (LMP: large microplastics, from 5000 to 100 µm; SMP-a: small microplastics from 100 to 10 µm; SMP-b: idem from 10 to 1 µm; NP: nanoplastics <1 µm) to marine and freshwater (blue shaded) organisms. PP: polypropylene; PA: polyamide; PE: polyethylene; PS: polystyrene; PHB: polyhydroxybutyrate; PET: polyethylene terephthalate; propiet. proprietary polymer of unknown composition.

| **Size Class** | **Composition** | **Test taxa** | **Test species** | **endpoint** | **parameter** | **TT (mg/L)** | **ref.** |
| --- | --- | --- | --- | --- | --- | --- | --- |
| LMP | virgin PP | bivalve | *Perna perna* | Embryo development | LOEC | 50000 | ^16^ |
| LMP | natural mix | fish cell lines | *Oncorhynchus mykiss* | Comet assay | LOEC | 100 | ^17^ |
| LMP | virgin PVC | microalgae | *Skeletonema costatum* | Population Growth | NOEC | 2000 | ^18^ |
| LMP | natural mix | fish | *O. melastigma* | Embryo-larval development | NOEC | 1000 | ^19^ |
| LMP | PA | crustaceans | *Gammarus fossarum* | Growth (4 weeks) | NOEC | 44.7 | ^20^ |
| LMP | virgin PE | crustaceans | *Daphnia magna* | Immobilization | NOEC | 400 | ^21^ |
| LMP | dyed PE | fish | *Cyprinodon variegatus* | Larval survival | NOEC | 250 | ^22^ |
| SMP-a | PS | fish | *Sebastes schlegelii* | Growth (14 d) | LOEC | 0.19 | ^23^ |
| SMP-a | virgin PS | crustaceans | *Calanus helgolandicus* | Reproductive output | LOEC | 0.31 | ^24^ |
| SMP-a | dyed PE | crustaceans | *Hyalella azteca* | Survival (10 d) | LOEC | 0.619 | ^25^ |
| SMP-a | dyed PE | echinoderms | *Tripneustes gratilla* | Larval growth | LOEC | 4.2 | ^26^ |
| SMP-a | PS-latex | bivalve | *Mytilus edulis* | Energy budget | NOEC | 4.55 | ^27^ |
| SMP-a | PE, PP, PS | crustaceans | *Palaemonetes pugio* | Adult mortality (96 h) | LOEC | 11 | ^28^ |
| SMP-a | PHB | crustaceans | *Gammarus fossarum* | Weight (4 weeks) | LOEC | 15.8 | ^29^ |
| SMP-a | virgin PE | microalgae | *P. tricornutum* | Population Growth (72 h) | NOEC | 25 | ^30^ |
| SMP-a | dyed PE | crustaceans | *Hyalella azteca* | Survival (10 d) | LOEC | 26 | ^25^ |
| SMP-a | PET fibres | crustaceans | *Daphnia magna* | Immobilization | EC_50_/3 | 33.33 | ^31^ |
| SMP-a | PVC | microalgae | *Skeletonema costatum* | Population Growth (96 h) | LOEC | 50 | ^32^ |
| SMP-a | PVC | microalgae | *Skeletonema costatum* | Population Growth (96 h) | NOEC | 100 | ^32^ |
| SMP-a | virgin PE | bivalve | *M. galloprovincialis* | Embryo development | NOEC | 100 | ^33^ |
| SMP-a | virgin PE | echinoderms | *Paracentrotus lividus* | Embryo development | NOEC | 100 | ^33^ |
| SMP-a | dyed PET | crustaceans | *Gammarus pulex* | Development (48 d) | NOEC | 122 | ^34^ |
| SMP-a |  | crustaceans | *Daphnia magna* | Immobilization | NOEC | 250 | ^35^ |
| SMP-a | dyed PE | crustaceans | *Gammarus duebeni* | Mobility (48 h) | NOEC | 391 | ^36^ |
| SMP-b | dyed PE | crustaceans | *Daphnia magna* | Reproduction (21 d) | LOEC | 0.005 | ^37^ |
| SMP-b | dyed PS | bivalve | *Crassostrea gigas* | Larval growth of progeny | LOEC | 0.01 | ^38^ |
| SMP-b | dyed, propriet. | crustaceans | *Daphnia magna* | Reproduction (21 d) | LOEC | 0.02 | ^39^ |
| SMP-b |  | fish | *Oryzias melastigma* | Larval weight (28 d) | LOEC | 0.02 | ^40^ |
| SMP-b | dyed PS | bivalve | *Pinctada margaritifera* | Energy budget | LOEC | 0.025 | ^41^ |
| SMP-b | PS | crustaceans | *Tigriopus japonicus* | Reprod. (chronic, 2 gener.) | LOEC | **0.1** | ^42^ |
| SMP-b | PS | gastropod | *Crepidula onyx* | Larval growth | LOEC | 0.25 | ^43^ |
| SMP-b | PS | crustaceans | *Centropages tipicus* | Ingestion rate | LOEC | 0.8 | ^44^ |
| SMP-b | dyed PE | crustaceans | *Daphnia magna* | Reproduction (21 d) | LOEC | 0.9 | ^45^ |
| SMP-b | virgin PVC | microalgae | *Skeletonema costatum* | Population Growth | LOEC | 1 | ^18^ |
| SMP-b | virgin PE | rotifer | *Brachionus plicatilis* | Mortality | LOEC | 1 | ^33^ |
| SMP-b | virgin PE | crustaceans | *Tigriopus fulvus* | Laval mortality | LOEC | **1** | ^33^ |
| SMP-b | PET | crustaceans | *Parvocalanus crassirostris* | Egg production | LOEC | **10** | ^46^ |
| SMP-b | virgin PE | crustaceans | *Daphnia magna* | Immobilization | EC_50_/3 | 19 | ^21^ |
| SMP-b | PS | rotifer | *Brachionus koreanus* | Growth | NOEC | 20 | ^47^ |
| SMP-b | PS | crustaceans | *Paracyclopina nana* | Fecundity | NOEC | 20 | ^48^ |
| SMP-b | PVC | bivalve | *Perna viridis* | Survival (90 d) | LOEC | 21.6 | ^49^ |
| SMP-b | virgin PE | microalgae | *P. tricornutum* | Population Growth (72 h) | NOEC | 25 | ^30^ |
| SMP-b | virgin PE | microalgae | *Isochrysis galbana* | Population Growth (72 h) | NOEC | 25 | ^50^ |
| SMP-b | dyed PS | echinoderms | *P. lividus* | Larval development | LOEC | **25** | ^51^ |
| SMP-b | dyed PS | ascidians | *Clavelina robusta* | Larval development | NOEC | **25** | ^51^ |
| SMP-b | dyed, propriet. | microalgae | *Tetraselmis chuii* | Population Growth (96 h) | NOEC | 41.5 | ^52^ |
| SMP-b | PVC | microalgae | *Skeletonema costatum* | Population Growth (96 h) | LOEC | 50 | ^32^ |
| SMP-b | PS | microalgae | *Chlorella pyrenoidosa* | Population Growth (96 h) | NOEC | 60 | ^53^ |
| SMP-b | virgin PE | bivalve | *M. galloprovincialis* | Embryo development | NOEC | 100 | ^33^ |
| SMP-b | virgin PE | echinoderms | *Paracentrotus lividus* | Embryo development | LOEC | 100 | ^33^ |
| SMP-b | PS | microalgae | *Dunaliella tertiolecta* | Population Growth (72 h) | NOEC | 250 | ^54^ |
| SMP-b |  | crustacean | *Daphnia magna* | Mortality (4 d) | NOEC | 400 | ^55^ |
| SMP-b | PS | bivalve | *Mytilus edulis* | Filtering rate | NOEC | 510 | ^56^ |
| NP | dyed PS | bivalve | *Crassostrea gigas* | Larval ingestion rate | LOEC | 0.0005 | ^57^ |
| NP | dyed PS | microalgae | *Dunaliella tertiolecta* | Population Growth (72 h) | LOEC | 0.001 | ^58^ |
| NP | PS | fish | *Sebastes schlegelii* | Growth (14 d) | LOEC | 0.19 | ^23^ |
| NP | PS | rotifer | *Brachionus koreanus* | Growth | LOEC | 0.1 | ^47^ |
| NP | PS | crustaceans | *Tigriopus japonicus* | Reprod. (chronic, 2 gener.) | LOEC | **0.1** | ^42^ |
| NP | PS | crustaceans | *Artemia salina* | Survival (14 d) | LOEC | 0.1 | ^59^ |
| NP | dyed PS | crustaceans | *Daphnia magna* | Feeding rate | LOEC | 1 | ^60^ |
| NP |  | crustacean | *Daphnia magna* | Survival (4 d) | EC_10_ | 1.54 | ^55^ |
| NP | dyed PS | echinoderms | *Paracentrotus lividus* | Embryo development | LOEC | 2,5 | ^61^ |
| NP | PS | microalgae | *Chlorella pyrenoidosa* | Population Growth (96 h) | IC_50_/3 | 3 | ^53^ |
| NP | PS | crustaceans | *Daphnia magna* | Immobilization | EC_50_/3 | 5.043 | ^62^ |
| NP | PS | crustaceans | *Paracyclopina nana* | Fecundity | LOEC | 10 | ^48^ |
| NP |  | crustaceans | *Daphnia magna* | immobilization | LOEC | 20 | ^63^ |
| NP | PS | microalgae | *Dunaliella tertiolecta* | Population Growth (72 h) | NOEC | 25 | ^54^ |


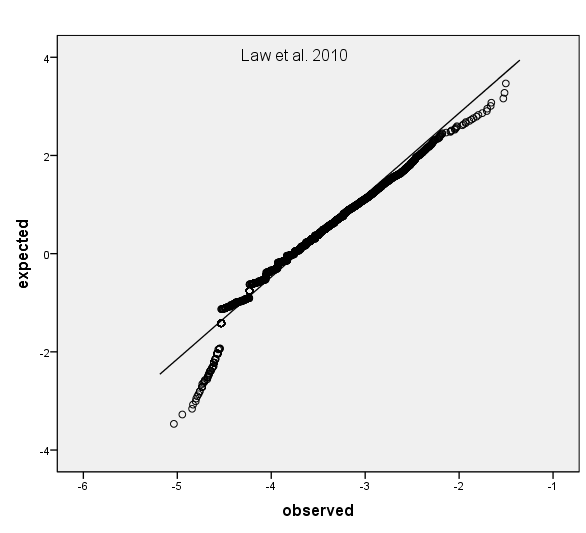


(a)


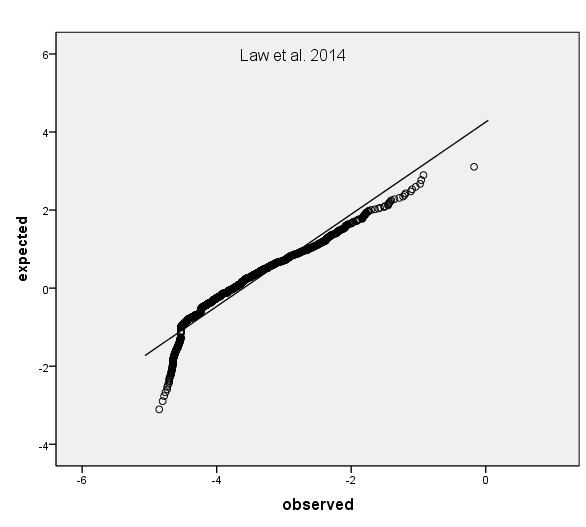


(b)

Fig S1a,b. Expected (according to a log-normal distribution) vs observed residuals for the Law et al. (2010) (above), and the Law et al. (2014) (below) datasets.


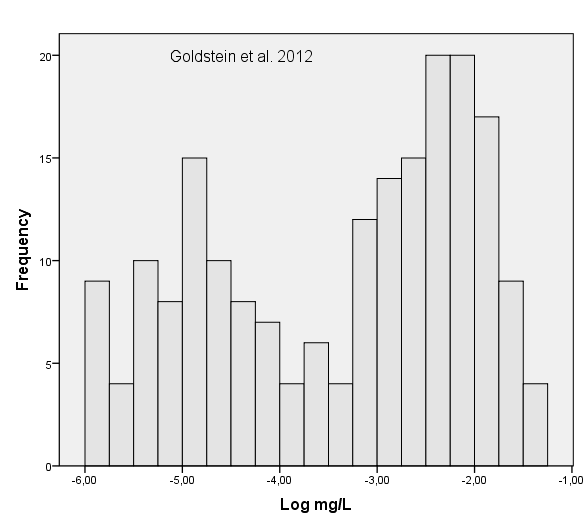


Fig. S2. Bimodal distribution of plastic density in the dataset reported by Goldstein et al. (2012).


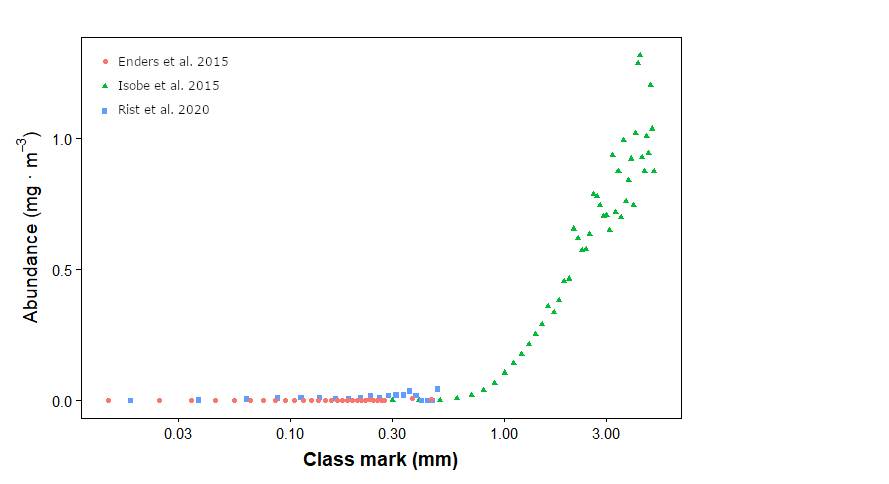


Fig. S3. Abundance of plastic particles (in mass units) according to their size. Note the low weight of particles below the 1 mm threshold in the overall abundance of microplastics. Data from Enders et al (2015), Isobe et al (2015) and Rist et al. (2020).

Supplementary references

1. Colton, J. B., Knapp, F. D. & Burns, B. R. Plastic particles in surface waters of the Northwestern Atlantic. *Science (80-. ).* **185**, 491–497 (1974).

2. Day, R. H. & Shaw, D. G. Patterns in the abundance of pelagic plastic and tar in the north pacific ocean, 1976-1985. *Mar. Pollut. Bull.* **18**, 311–316 (1987).

3. Day, R. H., Shaw, D. G. & Ignell, S. E. the Quantitative Distribution and Characteristics of Neuston Plastic in the North Pacific Ocean, 1985-88. *Proc. Second Int. Conf. Mar. Debris* 247–263 (1990) doi:10.1017/CBO9781107415324.004.

4. Ryan, P. G. The characteristics and distribution of plastic particles at the sea-surface off the southwestern Cape Province, South Africa. *Mar. Environ. Res.* **25**, 249–273 (1988).

5. Moore, C. J., Moore, S. L., Leecaster, M. K. & Weisberg, S. B. A comparison of plastic and plankton in the North Pacific Central Gyre. *Mar. Pollut. Bull.* **42**, 1297–1300 (2001).

6. Moore, C. J., Moore, S. L., Weisberg, S. B., Lattin, G. L. & Zellers, A. F. A comparison of neustonic plastic and zooplankton abundance in southern California’s coastal waters. *Mar. Pollut. Bull.* **44**, 1035–1038 (2002).

7. Yamashita, R., Tanimura, A. Floating plastic in the Kuroshio Current area, western North Pacific Ocean. *Mar. Pollut. Bull.* **54**, 464–488 (2007).

8. Zhao, S., Zhu, L., Wang, T. & Li, D. Suspended microplastics in the surface water of the Yangtze Estuary System, China: First observations on occurrence, distribution. *Mar. Pollut. Bull.* **86**, 562–568 (2014).

9. Song, Y. K. *et al.* Large accumulation of micro-sized synthetic polymer particles in the sea surface microlayer. *Environ. Sci. Technol.* **48**, 9014–9021 (2014).

10. Cózar, A. *et al.* Plastic debris in the open ocean. *Proc. Natl. Acad. Sci. U. S. A.* **111**, 10239–10244 (2014).

11. Ivar Do Sul, J. A., Costa, M. F. & Fillmann, G. Microplastics in the pelagic environment around oceanic islands of the western Tropical Atlantic Ocean. *Water. Air. Soil Pollut.* **225**, (2014).

12. Frias, J. P. G. L., Otero, V. & Sobral, P. Evidence of microplastics in samples of zooplankton from Portuguese coastal waters. *Mar. Environ. Res.* **95**, 89–95 (2014).

13. Isobe, A., Uchida, K., Tokai, T. & Iwasaki, S. East Asian seas: A hot spot of pelagic microplastics. *Mar. Pollut. Bull.* **101**, 618–623 (2015).

14. Faure, F. *et al.* An evaluation of surface micro- and mesoplastic pollution in pelagic ecosystems of the Western Mediterranean Sea. *Environ. Sci. Pollut. Res.* **22**, 12190–12197 (2015).

15. Gago, J., Henry, M. & Galgani, F. First observation on neustonic plastics in waters off NW Spain (spring 2013 and 2014). *Mar. Environ. Res.* **111**, 27–33 (2015).

16. Gandara e Silva, P. P., Nobre, C. R., Resaffe, P., Pereira, C. D. S. & Gusmão, F. Leachate from microplastics impairs larval development in brown mussels. *Water Res.* **106**, 364–370 (2016).

17. Pannetier, P. *et al.* Toxicity assessment of pollutants sorbed on environmental sample microplastics collected on beaches: Part I-adverse effects on fish cell line. *Environ. Pollut.* **248**, 1088–1097 (2019).

18. Zhang, C., Chen, X., Wang, J. & Tan, L. Toxic effects of microplastic on marine microalgae Skeletonema costatum: Interactions between microplastic and algae. *Environ. Pollut.* **220**, 1282–1288 (2017).

19. Pannetier, P. *et al.* Toxicity assessment of pollutants sorbed on environmental microplastics collected on beaches: Part II-adverse effects on Japanese medaka early life stages. *Environ. Pollut.* **248**, 1098–1107 (2019).

20. Blarer, P. & Burkhardt-Holm, P. Microplastics affect assimilation efficiency in the freshwater amphipod Gammarus fossarum. *Environ. Sci. Pollut. Res.* **23**, 23522–23532 (2016).

21. Rehse, S., Kloas, W. & Zarfl, C. Short-term exposure with high concentrations of pristine microplastic particles leads to immobilisation of Daphnia magna. *Chemosphere* **153**, 91–99 (2016).

22. Choi, J. S., Jung, Y. J., Hong, N. H., Hong, S. H. & Park, J. W. Toxicological effects of irregularly shaped and spherical microplastics in a marine teleost, the sheepshead minnow (Cyprinodon variegatus). *Mar. Pollut. Bull.* **129**, 231–240 (2018).

23. Yin, L. *et al.* Impacts of polystyrene microplastics on the behavior and metabolism in a marine demersal teleost, black rockfish (Sebastes schlegelii). *J. Hazard. Mater.* **380**, 120861 (2019).

24. Cole, M., Lindeque, P., Fileman, E., Halsband, C. & Galloway, T. S. The impact of polystyrene microplastics on feeding, function and fecundity in the marine copepod Calanus helgolandicus. *Environ. Sci. Technol.* **49**, 1130–1137 (2015).

25. Au, S. Y., Bruce, T. F., Bridges, W. C. & Klaine, S. J. Responses of Hyalella azteca to acute and chronic microplastic exposures. *Environ. Toxicol. Chem.* **34**, 2564–2572 (2015).

26. Kaposi, K. L., Mos, B., Kelaher, B. P. & Dworjanyn, S. A. Ingestion of microplastic has limited impact on a marine larva. *Environ. Sci. Technol.* **48**, 1638–1645 (2014).

27. Van Cauwenberghe, L., Claessens, M., Vandegehuchte, M. B. & Janssen, C. R. Microplastics are taken up by mussels (Mytilus edulis) and lugworms (Arenicola marina) living in natural habitats. *Environ. Pollut.* **199**, 10–17 (2015).

28. Gray, A. D. & Weinstein, J. E. Size- and shape-dependent effects of microplastic particles on adult daggerblade grass shrimp (Palaemonetes pugio). *Environ. Toxicol. Chem.* **36**, 3074–3080 (2017).

29. Straub, S., Hirsch, P. E. & Burkhardt-Holm, P. Biodegradable and petroleum-based microplastics do not differ in their ingestion and excretion but in their biological effects in a freshwater invertebrate Gammarus fossarum. *Int. J. Environ. Res. Public Health* **14**, (2017).

30. Gambardella, C. *et al.* Microplastics do not affect standard ecotoxicological endpoints in marine unicellular organisms. *Mar. Pollut. Bull.* **143**, 140–143 (2019).

31. Jemec, A., Horvat, P., Kunej, U., Bele, M. & Kržan, A. Uptake and effects of microplastic textile fibers on freshwater crustacean Daphnia magna. *Environ. Pollut.* **219**, 201–209 (2016).

32. Zhu, Z. *et al.* Joint toxicity of microplastics with triclosan to marine microalgae Skeletonema costatum. *Environ. Pollut.* **246**, 509–517 (2019).

33. Beiras, R. *et al.* Ingestion and contact with polyethylene microplastics does not cause acute toxicity on marine zooplankton. *J. Hazard. Mater.* **360**, 452–460 (2018).

34. Weber, A., Scherer, C., Brennholt, N., Reifferscheid, G. & Wagner, M. PET microplastics do not negatively affect the survival, development, metabolism and feeding activity of the freshwater invertebrate Gammarus pulex. *Environ. Pollut.* **234**, 181–189 (2018).

35. Rehse, S., Kloas, W. & Zarfl, C. Microplastics reduce short-term effects of environmental contaminants. Part I: Effects of bisphenol a on freshwater zooplankton are lower in presence of polyamide particles. *Int. J. Environ. Res. Public Health* **15**, (2018).

36. Mateos-Cárdenas, A. *et al.* Polyethylene microplastics adhere to Lemna minor (L.), yet have no effects on plant growth or feeding by Gammarus duebeni (Lillj.). *Sci. Total Environ.* **689**, 413–421 (2019).

37. Jaikumar, G., Brun, N. R., Vijver, M. G. & Bosker, T. Reproductive toxicity of primary and secondary microplastics to three cladocerans during chronic exposure. *Environ. Pollut.* **249**, 638–646 (2019).

38. Sussarellu, R. *et al.* Oyster reproduction is affected by exposure to polystyrene microplastics. *Proc. Natl. Acad. Sci. U. S. A.* **113**, 2430–2435 (2016).

39. Pacheco, A., Martins, A. & Guilhermino, L. Toxicological interactions induced by chronic exposure to gold nanoparticles and microplastics mixtures in Daphnia magna. *Sci. Total Environ.* **628**–**629**, 474–483 (2018).

40. Li, Y. *et al.* Low level of polystyrene microplastics decreases early developmental toxicity of phenanthrene on marine medaka (Oryzias melastigma). *J. Hazard. Mater.* **385**, 121586 (2020).

41. Gardon, T., Reisser, C., Soyez, C., Quillien, V. & Le Moullac, G. Microplastics Affect Energy Balance and Gametogenesis in the Pearl Oyster Pinctada margaritifera. *Environ. Sci. Technol.* **52**, 5277–5286 (2018).

42. Lee, K. W., Shim, W. J., Kwon, O. Y. & Kang, J. H. Size-dependent effects of micro polystyrene particles in the marine copepod tigriopus japonicus. *Environ. Sci. Technol.* **47**, 11278–11283 (2013).

43. Lo, H. K. A. & Chan, K. Y. K. Negative effects of microplastic exposure on growth and development of Crepidula onyx. *Environ. Pollut.* **233**, 588–595 (2018).

44. Cole, M. *et al.* Cole M. Microplastic ingestion by Zooplankton. ES&T 2013. *Environ. Sci. Technol.* **47**, 6646–6655 (2013).

45. Ogonowski, M., Schür, C., Jarsén, Å. & Gorokhova, E. The effects of natural and anthropogenic microparticles on individual fitness in daphnia magna. *PLoS One* **11**, 1–20 (2016).

46. Heindler, F. M. *et al.* Toxic effects of polyethylene terephthalate microparticles and Di(2-ethylhexyl)phthalate on the calanoid copepod, Parvocalanus crassirostris. *Ecotoxicol. Environ. Saf.* **141**, 298–305 (2017).

47. Jeong, C. B. *et al.* Microplastic Size-Dependent Toxicity, Oxidative Stress Induction, and p-JNK and p-p38 Activation in the Monogonont Rotifer (Brachionus koreanus). *Environ. Sci. Technol.* **50**, 8849–8857 (2016).

48. Jeong, C. B. *et al.* Adverse effects of microplastics and oxidative stress-induced MAPK/Nrf2 pathway-mediated defense mechanisms in the marine copepod Paracyclopina nana. *Sci. Rep.* **7**, 1–11 (2017).

49. Rist, S. E. *et al.* Suspended micro-sized PVC particles impair the performance and decrease survival in the Asian green mussel Perna viridis. *Mar. Pollut. Bull.* **111**, 213–220 (2016).

50. Garrido, S., Linares, M., Campillo, J. A. & Albentosa, M. Effect of microplastics on the toxicity of chlorpyrifos to the microalgae Isochrysis galbana, clone t-ISO. *Ecotoxicol. Environ. Saf.* **173**, 103–109 (2019).

51. Messinetti, S., Mercurio, S., Parolini, M., Sugni, M. & Pennati, R. Effects of polystyrene microplastics on early stages of two marine invertebrates with different feeding strategies. *Environ. Pollut.* **237**, 1080–1087 (2018).

52. Prata, J. C., Lavorante, B. R. B. O., Maria da, M. da C. & Guilhermino, L. Influence of microplastics on the toxicity of the pharmaceuticals procainamide and doxycycline on the marine microalgae Tetraselmis chuii. *Aquat. Toxicol.* **197**, 143–152 (2018).

53. Yi, X. *et al.* Combined effect of polystyrene plastics and triphenyltin chloride on the green algae Chlorella pyrenoidosa. *Environ. Sci. Pollut. Res.* **26**, 15011–15018 (2019).

54. Sjollema, S. B., Redondo-Hasselerharm, P., Leslie, H. A., Kraak, M. H. S. & Vethaak, A. D. Do plastic particles affect microalgal photosynthesis and growth? *Aquat. Toxicol.* **170**, 259–261 (2016).

55. Fadare, O. O. *et al.* Humic acid alleviates the toxicity of polystyrene nanoplastic particles to Daphnia magna. *Environ. Sci. Nano* **6**, 1466–1477 (2019).

56. Browne, M. A., Dissanayake, A., Galloway, T. S., Lowe, D. M. & Thompson, R. C. Ingested microscopic plastic translocates to the circulatory system of the mussel, Mytilus edulis (L.). *Environ. Sci. Technol.* **42**, 5026–5031 (2008).

57. Cole, M. & Galloway, T. S. Ingestion of Nanoplastics and Microplastics by Pacific Oyster Larvae. *Environ. Sci. Technol.* **49**, 14625–14632 (2015).

58. Gambardella, C. *et al.* Ecotoxicological effects of polystyrene microbeads in a battery of marine organisms belonging to different trophic levels. *Mar. Environ. Res.* **141**, 313–321 (2018).

59. Varó, I. *et al.* Time-dependent effects of polystyrene nanoparticles in brine shrimp Artemia franciscana at physiological, biochemical and molecular levels. *Sci. Total Environ.* **675**, 570–580 (2019).

60. Rist, S., Baun, A. & Hartmann, N. B. Ingestion of micro- and nanoplastics in Daphnia magna – Quantification of body burdens and assessment of feeding rates and reproduction. *Environ. Pollut.* **228**, 398–407 (2017).

61. Della Torre, C. *et al.* Accumulation and embryotoxicity of polystyrene nanoparticles at early stage of development of sea urchin embryos Paracentrotus lividus. *Environ. Sci. Technol.* **48**, 12302–12311 (2014).

62. Ma, Y. *et al.* Effects of nanoplastics and microplastics on toxicity, bioaccumulation, and environmental fate of phenanthrene in fresh water. *Environ. Pollut.* **219**, 166–173 (2016).

63. Kim, D., Chae, Y. & An, Y. J. Mixture Toxicity of Nickel and Microplastics with Different Functional Groups on Daphnia magna. *Environ. Sci. Technol.* **51**, 12852–12858 (2017).
